# Profiling the Biophysical Developability Properties of Common IgG1 Fc Effector Silencing Variants

**DOI:** 10.3390/antib12030054

**Published:** 2023-08-22

**Authors:** Robert Pejchal, Anthony B. Cooper, Michael E. Brown, Maximiliano Vásquez, Eric M. Krauland

**Affiliations:** 1 Adimab LLC, Lebanon, NH 03766, USA; michael.brown@adimab.com (M.E.B.); max.vasquez@adimab.com (M.V.); eric.krauland@adimab.com (E.M.K.); 2 Aclys Bio, Lebanon, NH 03766, USA; tony@aclys.bio

**Keywords:** antibody, CD3, T-cell engager, Fcγ receptor, Fc effector silencing, C1q

## Abstract

Therapeutic antibodies represent the most significant modality in biologics, with around 150 approved drugs on the market. In addition to specific target binding mediated by the variable fragments (Fvs) of the heavy and light chains, antibodies possess effector functions through binding of the constant region (Fc) to Fcγ receptors (FcγR), which allow immune cells to attack and kill target cells using a variety of mechanisms. However, for some applications, including T-cell-engaging bispecifics, this effector function is typically undesired. Mutations within the lower hinge and the second constant domain (CH2) of IgG1 that comprise the FcγR binding interface reduce or eliminate effector function (“Fc silencing”) while retaining binding to the neonatal Fc receptor (FcRn), important for normal antibody pharmacokinetics (PKs). Comprehensive profiling of biophysical developability properties would benefit the choice of constant region variants for development. Here, we produce a large panel of representative mutations previously described in the literature and in many cases in clinical or approved molecules, generate select combinations thereof, and characterize their binding and biophysical properties. We find that some commonly used CH2 mutations, including D265A and P331S, are effective in reducing binding to FcγR but significantly reduce stability, promoting aggregation, particularly under acidic conditions commonly employed in manufacturing. We highlight mutation sets that are particularly effective for eliminating Fc effector function with the retention of WT-like stability, including L234A, L235A, and S267K (LALA-S267K), L234A, L235E, and S267K (LALE-S267K), L234A, L235A, and P329A (LALA-P329A), and L234A, L235E, and P329G (LALE-P329G).

## 1. Introduction

Therapeutic antibodies are widely used in the treatment of various diseases, including cancer, autoimmune disorders, and infectious diseases [1]. Antibodies, which belong to a class of therapeutic drugs termed biologics, have favorable properties including highly specific and high-affinity binding to targets (i.e., low or no specific off-target binding), a long serum half-life, and the ability to trigger immune responses, termed effector functions. However, these effector functions can also cause unintended adverse effects, including inflammation, tissue damage, and cytotoxicity. In particular, T cell-engaging bispecifics benefit from abrogation of effector functions [2]. Therefore, there has long been interest in developing antibodies with reduced effector functions that maintain their antigen-binding capacity and other important drug-like properties [3]. This approach is known as “effector function modulation” or “silencing” and is achieved through various strategies, such as modification of the Fc region, alteration of glycosylation patterns, and engineering of the antibody structure. These modifications can significantly impact the pharmacokinetics and pharmacodynamics of antibodies, including their serum half-life, clearance, and tissue distribution. Thus, reducing effector function in IgG1 antibodies has the potential to enhance their safety and efficacy in the clinical setting.

Human immune cells express four activating FcγRs: FcγRI (CD64), FcγRIIa (CD32a), FcγRIIc (CD32c), and FcγRIIIa (CD16a) [4], which bind to the hinge and upper CH2 domains of Fc [5]. These receptors mediate antibody-dependent cellular cytotoxicity (ADCC) and antibody-dependent cell-mediated phagocytosis (ADCP) [6]. IgG recycling, which contributes to a long half-life, is mediated by the neonatal Fc receptor (FcRn) [7]. FcRn binding to Fc is pH dependent, with stronger binding observed under the acidic conditions (<pH 6.5) present in the endosome [8]. The Fc also engages the complement system, a component of the innate immune pattern recognition system that functions to destroy targeted cells through activation of the C1 complex, which triggers a cascade that leads to cell lysis known as complement-dependent cytotoxicity (CDC). In the classical pathway, C1 activation is initiated by the binding of the complement C1q heterotrimer to the Fc, which recruits the proteases C1r and C1s to form the C1 complex [9]. C1 then converts to C3, which ultimately forms C3a and C3b. Finally, of importance to antibody manufacturing at a large scale is the ability to bind protein A (ProA), a bacterial protein with nM affinity for the Fc. Pro affinity chromatography is the first step in the purification of an antibody from culture supernatant [10].

**Figure 1:**
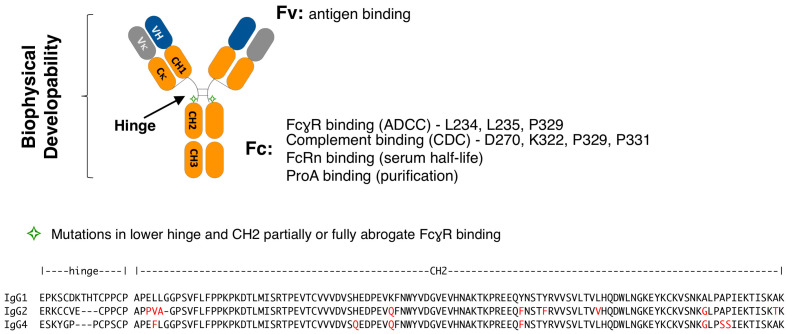
(**top**) Schematic representation of IgG1. The heavy chain is composed of the variable heavy (VH) and constant domains 1, 2, and 3 (CH1, CH2, and CH3, respectively). A disulfide bonded hinge region between CH1 to CH2 flexibly links these domains. The light chain is composed of a variable light and constant light domains of isotype kappa (κ) or lambda (λ). Antigen binding is mediated by the VH and Vκ (for a kappa isotype antibody, as shown), together referred to as the variable fragment (Fv). The constant fragment (Fc) mediates binding to FcγR, complement, FcRn, and ProA. Residues strongly associated with binding to FcγR and complement are listed. (**bottom**) The hinge and CH2 domain sequences of the IgG1, IgG2, and IgG4 subclasses are shown, with differences in amino acids indicated in red font and differences in length marked by dashes.

IgG subclasses differ in their binding affinity to FcγRs and complement [11]. As such, IgG2 and IgG4 have been used to reduce Fc effector function when ADCC and CDC are not desired [12]. However, one drawback of this strategy is that IgG2 and IgG4 show increased sensitivity to acid-induced aggregation [13], with IgG4 having a significantly lower melting temperature (Tm) and higher onset pH threshold for aggregation [14,15]. An ideal silent IgG1 Fc would possess the following properties: no binding to activating FcγR and C1q, retention of binding to FcRn and Protein A (ProA), and biophysical properties non-inferior to wild-type IgG1 (Figure 1). The mapping of FcγR-Fc contact residues in IgG1 and the availability of crystal structures has provided functional insights and atomic level description that comprise a comprehensive picture of relevant molecular interactions that enable rational engineering approaches [16,17,18,19,20]. These structure–function studies establish the lower hinge, particularly L234 and L235, and P329, as the primary binding site of FcγRs [17,21,22]. Additional secondary interactions are mediated by residues in the vicinity of the CH2 glycan at N297, which also makes some contacts (EU numbering) [17,21,22,23,24,25]. The binding site for C1q overlaps that of FcγR [26], whereas the binding sites for FcRn and Protein A localize to the CH2-CH3 junction [27,28]. CH2 residues D270, K322, P329, and P331 have been identified as critical to the interaction between human IgG1 antibodies and C1q [29,30,31].

Early efforts to generate a silence Fc began with modification of the lower hinge, utilizing mutations L234A/L235A (LALA) [32] and L234A/L235A/G237A (LALAGA) [33], which showed a reduction in but not the complete elimination of effector functions. Subsequent combinations of hinge and CH2 modifications lead to a significant reduction in or the total elimination of function with preservation of FcRn and ProA binding. These mutation sets can be conceptually divided into approaches that retain the N297 glycan, exemplified by P331S [34], L234A/L235A-P329G (LALA-PG) [35], L234F/L235E-P331S (LFLE-PS) [22], L234F/L235E-D265A (LFLE-DA) [36], and others [37,38,39], and those that remove it, such as N297A [40], N297G [41], R292C/N297G/V302C [42], and others.

In this work, we set out to characterize the binding and biophysical developability of commonly used Fc silencing mutation sets. We produced and evaluated mutations in the lower hinge and CH2 of the IgG1 anti-CD3ε antibody ADI-26906 [43]. We observed that S267K, P329G, and P329A mutations combined with lower hinge mutations LALA and LALE completely eliminated binding to FcγR under the conditions of the assays and retained WT-like biophysical properties. In contrast, D265A and P331S combined with the same hinge mutations increase acid-induced aggregation. Sensitivity to pH stress in general was observed to correlate with reduced CH2 melting temperature (Tm).

## 2. Materials and Methods

### 2.1. Polyspecificity Reagent (PSR) Binding Assay

Antibody PSR binding was carried out following previously described methods [44,45,46]. In summary, CHO cells were used to extract soluble membrane protein (SMP) and soluble cytosolic protein (SCP) fractions, which were subsequently biotinylated using the NHS-LC-Biotin reagent from Thermo Fisher Scientific (Waltham, MA, USA) (#A39257). Yeast-presented IgGs were then incubated with a 1:10 dilution of the biotinylated SMP and SCP stocks for 20 min at ice-cold temperatures, washed twice with PBSF [47], and stained with a secondary labeling mix composed of ExtrAvidin-R-PE from Sigma-Aldrich (St. Louis, MO, USA) (#E4011), anti-human LC-FITC from Southern Biotech (Birmingham, AL, USA) (#2062-02), and propidium iodide from Sigma-Aldrich (#11348639001) for 15 min on ice. The cells were subsequently washed with PBSF and suspended in PBSF for flow cytometric analysis on a BD FACS Canto II instrument from BD Biosciences (San Jose, CA, USA). The binding mean fluorescence intensity (MFI) was determined with a flow cytometry analyzer and normalized to a score ranging from 0 to 1 using three control antibodies that indicate low, medium, and high binding to the PSR reagent.

### 2.2. Affinity-Capture Self-Interaction Nanoparticle Spectroscopy (AC-SINS)

AC-SINS is used to assess self-interaction propensity [48,49]. In brief, gold nanoparticles (Ted Pella Inc. (Redding, CA, USA) 15705) were coated with anti-human goat IgG Fc (Jackson ImmunoResearch (West Grove, PA, USA) 109-005-098) and incubated with IgGs for 60 min. Subsequently, the wavelength shift was evaluated using Molecular Devices SpectraMax M2 with SoftMax Pro6 software (San Jose, CA, USA). Elevated levels of particle aggregates were inferred when self-interacting clones exhibited a higher wavelength shift away from the PBS sample.

### 2.3. Hydrophobic Interaction Chromatography (HIC)

HIC is a chromatography assay used to characterize the hydrophobicity of antibodies [50]. In summary, 5 μg of IgG samples (1 mg/mL) was mixed with a mobile phase A solution (1.8 M ammonium sulfate and 0.1 M sodium phosphate at pH 6.5) to achieve a final ammonium sulfate concentration of approximately 1 M prior to analysis. The analysis was performed using a Sepax Proteomix HIC butyl-NP5 column with a linear gradient of mobile phase A and mobile phase B solution (0.1 M sodium phosphate, pH 6.5) over 20 min at a flow rate of 1 mL/min. UV absorbance at 280 nm was monitored during the analysis.

### 2.4. Size Exclusion Chromatography (SEC)

SEC is used to characterize the purity and quality of antibody samples. In brief, 2 μg of IgG in HBS buffer (25 mM HEPES, 150 mM sodium chloride, pH 7.3) was applied over a TSKgel SuperSW mAb HTP column (TOSOH Bioscience (South San Francisco, CA, USA) catalog number 22855) on an Agilent 1100 high-pressure liquid chromatography (HPLC) system. Absorbance at 280 nm wavelength was used for the chromatography profile, and elution peaks profiling was carried out using ChemStation software version 3.03.2.

### 2.5. pH 3.5 Stress SEC

To prepare the IgG samples for pH 3.5 stress and size exclusion chromatography, the antibodies were initially concentrated using spin column concentrators to a concentration of around 20 mg/mL in the original HEPES-buffered saline. Next, equal volumes of each IgG sample (usually 13–50 μL) were buffer exchanged using Zeba columns from Thermo Scientific into either PBS at pH 7.4 or 200 mM acetic buffer at pH 3.5 with 50 mM of NaCl. The buffer exchanged samples were adjusted to a final concentration of 15 mg/mL using the corresponding buffer and incubated at room temperature for 1 h. After the incubation period, samples in both buffers were diluted to a final concentration of 1 mg/mL by adding in 14× volumes of PBS and then refrigerated. Finally, size exclusion chromatography was performed immediately on the samples.

### 2.6. Thermal Melting (Tm) Measurements by Differential Scanning Fluorescence (DSF)

Tm was determined by utilizing the CFX96 Real-Time System from BioRad (Hercules, CA, USA), as previously described [51]. In summary, 20 μL of 1 mg/mL sample was mixed with 10 μL of 20× SYPRO orange, and the plate was scanned from 40 °C to 95 °C at a rate of 0.5 °C/2 min. The Fc Tm was assigned by analyzing the first derivative of the raw data through BioRad analysis software (Hercules, CA, USA).

### 2.7. Profiling FcγR Present on Human Leukemia Monocytic Line THP-1

Detection antibodies were sourced from the following commercial vendors: anti-FcγRI (FCGR1A/FCGRIB/CD64) clone 10.1 was sourced from BD Biosciences, anti-FcγRII (FCGR2A/FCGR2B/CD32) clone IV.3 was sourced from Invitrogen (Waltham, MA, USA), and anti-FcγRIII (FCGR3A/CD16) clone 3G8 was sourced from BD Biosciences.

THP-1 cells were sourced from DSMZ (Braunschweig, Germany) (catalog number ACC 16) and cultured in 90% RPMI 1640 media supplemented with 10% fetal bovine serum (FBS), according to the manufacturer’s recommendations. PC3 cells were obtained from Sigma-Aldrich (catalog number 90112714) and cultured in Coons Modified Ham’s F12 supplemented with 2 mM Glutamine and 7% FBS.

### 2.8. Assessing the Binding of Variant IgG1 Antibodies to THP-1 under Highly-Avid Conditions

WT IgG1 and modified anti-CD3ε antibody ADI-26906 were tested for binding to human THP-1 and PC3 cells. In brief, 100 nM IgG was incubated with the cells for 30 min on ice. Following two washes with PBSF (PBS + 0.1% bovine serum albumin (BSA)), IgGs were detected with goat anti-Human IgG R-PE secondary reagent (Southern Biotech, #2040–09) and analyzed by flow cytometry using a BD FACSCanto II (BD Bioscience). FCS Express software (De Novo Software, version 5) was used for data analysis, and binding was expressed as median fluorescent intensity (MFI).

Increasing the sensitivity of binding to THP-1 was accomplished by precomplexing variant antibodies with a CD3ε N-terminal peptide (residues 21–47) first conjugated to BSA (hereafter referred to as CD3ε-BSA, procured from New England Peptide (Gardner, MA, USA). Approximately 100 nM of each Fc variant antibody was incubated with 10 nM CD3ε-BSA at room temperature for 20 min. The samples were then mixed with THP-1 cells (200,000 cells per well) and incubated on ice for 30 min. Following three washes with PBSF, the cells were incubated with 100 μL of goat anti-human IgG PE antibody (Southern Biotech) at a dilution of 1:200 for 20 min at room temperature. The cells were then washed twice with PBSF and resuspended in 100 μL PBSF and then analyzed on a FACS Canto (BD Biosciences). Median fluorescence intensity (MFI) was recorded.

### 2.9. Dynal Bead Assay for the Detection of Complement Activation by IgG

A biotinylated b-CD3ε peptide was loaded to streptavidin Dyna Beads for 15 min at room temperature (RT) in PBSF. Following two washes, the beads were incubated with excess antibody to saturation, for 15 min in PBSF. The beads were then incubated with either C1q alone or with C1q and 100 μL of human complement C3 (Sigma) for 25 min at RT in PBSF. Finally, the beads were washed and labeled with anti-C1q APC and anti-C3 PE.

### 2.10. Biolayer Interferometry (BLI) Binding Asesssment of Variant Antibodies

Biolayer interferometry kinetic measurements were acquired at 25 °C with a ForteBio Octet HTX instrument (Sartorius, Bohemia, NY, USA). The reagents were formulated into a running buffer (PBSF) of phosphate-buffered saline with 0.1% IgG-free bovine serum albumin.

Human or cyno CD3 kinetics. The IgGs (100 nM) were first captured to anti-human IgG Fc capture (AHC) sensors (Sartorius, Bohemia, NY, USA) to a response level of 0.5–1.5 nm and then allowed to stand in PBSF for a minimum of 15 min before proceeding to the kinetic measurements. The kinetic measurements began with a short (60 s) baseline dip into PBSF before exposing (180 s) the IgG loaded sensors to human or cyno CD3 (100 nM). This was immediately followed by a dip (180 s) into PBSF to measure the rate of dissociation of the antibody–CD3 complex.

Protein A kinetics. Protein A sensors (Sartorius, Bohemia, NY, USA) were allowed to stand in PBSF for a minimum of 15 min before proceeding to the kinetic measurements. The kinetic measurements began with a short (60 s) baseline dip into PBSF before exposing (180 s) the IgG (concentrations ranged between 292 and 1100 nM) to blank sensor tips. This was immediately followed by a dip (180 s) into PBSF to measure the rate of dissociation of the IgG from the Protein A sensor tip.

All data were fit to a 1:1 binding model using ForteBio Data Analysis Software version 11.1.3.10.

### 2.11. Biacore Surface Plasmon Resonance (SPR) Binding Assessment of Variant Antibodies

Kinetic analysis was conducted at 25 °C in an HBS-EP+ running buffer system (10 mM HEPES pH 7.4, 150 mM NaCl, 3 mM EDTA, 0.05% Surfactant P20) using a Biacore 8K or 8K+ optical biosensor (Cytiva USA, Marlborough, MA, USA). The sample compartment was maintained at 10 °C for the duration of each experiment.

Human C1q kinetics. A goat anti-human Fc antibody (Jackson ImmunoResearch, Cat# 109-005-008) was covalently coupled to flow cells 1 and 2 of a CM5 sensor chip surface via standard amine coupling (1:1 EDC:NHS) and then blocked with ethanolamine. The antibodies were then captured (~1250 RU) to flow cell 2. A 100 nM solution of human C1q was injected (180 s, 30 µL/min), over both flow cells 1 and 2. Several blank buffer samples were also injected (30 µL/min) over flow cells 1 and 2 for the purpose of reference surface subtraction. Dissociation of the antigen from the sensor surface was monitored for 300 s at 30 µL/min. All surfaces were regenerated with three injections (15 s, 30 µL/min) of 10 mM glycine, pH 1.5.

Human FcγR kinetics. A goat anti-human Fc antibody (Jackson ImmunoResearch, Cat# 109-005-008) was covalently coupled to flow cells 1 and 2 of a CM5 sensor chip surface via standard amine coupling (1:1 EDC:NHS) and then blocked with ethanolamine. The antibodies were then captured (400–425 RU) to flow cell 2. For experiments with human FcγRI, a 100 nM solution of FcγR was injected (30 µL/min) over flow cells 1 and 2. For experiments with all other FcγRs, a 1000 nM solution of FcγR was injected (30 µL/min) over flow cells 1 and 2. Several blank buffer samples were injected over flow cells 1 and 2 for the purpose of reference surface subtraction. Dissociation of the antigen from the sensor surface was monitored for 300 s at 30 µL/min. All surfaces were regenerated with two injections (15 s, 30 µL/min) of 10 mM glycine, pH 1.5.

Human FcRn kinetics. A biotinylated CD3 peptide was captured (45 RU) to flow cell 1 of a streptavidin (SA) sensor (Cytiva USA, Marlborough, MA, USA) and after a short equilibration period (>60 min), IgG (100 nM) was then captured (400 RU) to flow cell 2. A solution of human FcRn (100 nM) was exposed (180 s, 30 µL/min) to flow cells 1 and 2. Several blank buffer samples were also injected (30 µL/min) over flow cells 1 and 2 for the purpose of reference surface subtraction. Dissociation of human FcRn from the IgG was monitored for 180 s. All surfaces were regenerated with two injections (15 s, 30 µL/min) of 10 mM glycine, pH 1.5.

Data processing and fitting. The sensograms were cropped to include only the association and dissociation steps. These cropped data were subsequently aligned, double reference subtracted, and then non-linear least squares fit to a 1:1 binding model using Biacore Insight Evaluation software version 3.0.11.15423 [52].

## 3. Results

### 3.1. Lower Hinge Variants

Lower hinge substitutions were incorporated into the anti-CD3 IgG1 antibody ADI-26906. These substitutions were derived from literature, clinical, and marketed molecules: L234A/L235A (LALA) [32], L234F/L235E (LFLE) [22], L234A/L235E (LALE) [39], L235A/G237A (LAGA1), L234A/G237A (LAGA2), and L234A/L235A/G237A (LALAGA) [33]. Derivatives representing a charge flip (L235K) were also explored, including L234A/L235K (LALK), L234F/L235K (LFLK), and L234E/L235K (LELK), to assess the impact of a positive charge in the lower hinge.

The mutation sets are enumerated in Table 1. All variants were produced in HEK cells by transient transfection and purified by ProA. Biophysical properties were profiled using a panel of established assays. In brief, polyspecificity reagent (PSR) binding was characterized by a FACS-based assay and reported as a normalized binding score [3,45]. Self-interaction propensity was characterized by affinity-capture self-interaction nanoparticle spectroscopy (AC-SINS) and reported as maximum wavelength shift (Δmaxλ) [49]. Hydrophobicity was characterized by hydrophobic interaction chromatography (HIC) and reported as retention time (min) [50]. Finally, antibody quality was characterized by size-exclusion chromatography (SEC) and reported as monomer content (%). Overall, the biophysical properties profiled were largely unaffected by lower hinge substitutions (Table 1). However, a significant impact was observed for L235K-containing hinge variants, which appear to slightly increase Fc Tm but negatively impact low pH stability, as assessed by the pH 3.5 stress assay.

The lower hinge variants were tested for binding to FcγR using a FACS-based cell binding assay. THP-1 cells express FcγRI and FcγRII (Figure S1), and binding of WT IgG1 is readily observable under standard conditions of 100 nM IgG labeling. All of the lower hinge variants reduced binding by approximately one order of magnitude, comparable to the level of WT IgG2 and in agreement with expectations [11]. No impact on binding of the variants to CD3δε was observed by Octet BLI (Table S1).

### 3.2. Lower Hinge and CH2 Variants

CH2 substitutions were incorporated into LALA and LALE lower hinge variants of the anti-CD3 IgG1 antibody ADI-26906. The mutation sets are enumerated in Table 2. As with the lower hinge, mutations were derived from literature, clinical, and marketed molecules. The panel comprises D265A [53], S267K [37,54], H268Q [55], D270A [29], Y296F [56], L309A [19], L309V, K322A [39], A327G [37], L328A [57], P329A [29], P329G [35], A330S [58], A330S/P331S [32], and P331S [22]. Variants H268Q, L309V, A330S and P331S are derived from IgG4.

**Figure 2:**
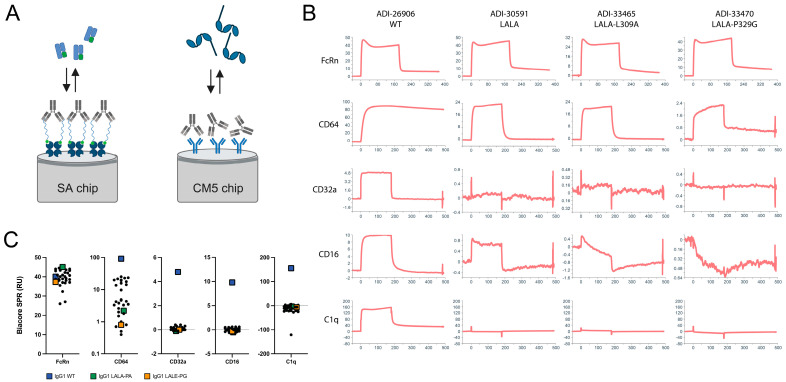
Profiling binding of IgG1 WT and variants to FcRn, CD64 (FcγRI), CD32a (FcγRII), CD16 (FcγRIII), and C1q by Biacore SPR. (**A**) Schematic representation of assay format for FcRn, FcγR, and C1q, created with BioRender. (**B**) Representative Biacore SPR sensograms. The *Y*-axis range is floated to show details of the association and dissociation phases. (**C**) Grouped dot plots of SPR response units for the panel of hinge and CH2 mutant variants, with WT IgG1 indicated by the blue squares, IgG1 LALA-P329A indicated by the green squares, and IgG1 LALE-P329G indicated by the orange squares.

Biacore SPR kinetics measurements were used as the first screen to evaluate affinity for FcRn, FcγRs, and C1q (Figure 2 and Table 2). For the FcRn experiments, the antibody variants were oriented by binding to the immobilized biotinylated CD3 peptide on the streptavidin sensor chip surface (Figure 2A). FcRn binding was retained in most variants. Only L309A negatively affected affinity for FcRn, as previously reported [19], whereas L309V has no effect. Binding to FcγRs utilized a different format: the capture of antibody variants with a goat anti-human Fc antibody immobilized to the CM5 sensor chip surface through amine coupling (Figure 2A). CD64 was most strongly reduced by D265A, S267K, P329G, and P329A, with LALE offering slightly more reduction than LALA (Table 2). This pattern is similar for CD32a and CD16; however, the low binding response for these weaker interactions makes the relative loss of binding more difficult to assess. C1q binding was observed only for WT IgG1, with both LALA and LALE hinge mutations ablating binding under the conditions of the assay.

**Figure 3:**
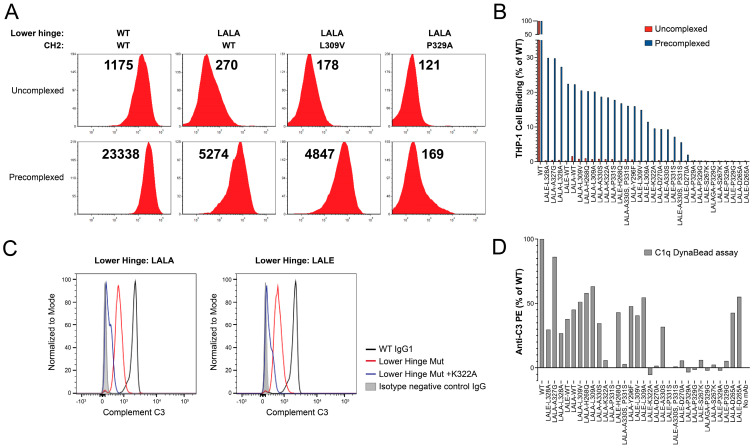
Elimination of binding to FcγRI and FcγRII presenting THP-1 cells. The THP-1 cells were confirmed to express cell surface FcγRI and FcγRII (Supplementary Figure S1). (**A**) Representative FACS profiles for WT, LALA, LALA-L309V, and LALA-P329A variants under uncomplexed and pre-complexed conditions. Median fluorescence intensity (MFI) values are shown above each profile. (**B**) Waterfall bar graph of MFI values collected for the 100 nM ADI-26906 antibody and variants without pre-complexing (red) or with pre-complexing to CD3ε-BSA (blue), conferring multivalent binding, as a percentage of WT. While hinge mutations alone are sufficient to reduce antibody binding under standard assay conditions, only combinations with some, but not all, previously described CH2 mutations are able to eliminate binding under highly avid conditions mediated by pre-complexing to CD3-BSA. (**C**) Representative C3 binding profiles for the C1q Dyna Bead immune complex assay. Clear separation of C3 activation by WT, lower hinge variants, and lower hinge and K332A combination variants is observed. (**D**) Bar graph of C3 activation by C1q bound by the antibody immune complex-coated Dyna Beads. The order of antibody variants is the same as in panel (**B**).

In order to assess binding to FcγRs on the surface of the cells, the THP-1 cells were labeled under standard (uncomplexed IgG) and highly avid conditions (Figure 3 and Table 2). Pre-complexing of the antibody with BSA multiply conjugated with the CD3 peptide (CD3-BSA PC) produced an approximately dodecavalent immune complex for the high avidity labeling of THP-1 cells. Both LALA and LALE were sufficient to reduce binding to THP-1 cells close to background under standard conditions, but these lower hinge variants still retained significant binding under the more sensitive antibody immune complex condition (Figure 3A,B and Table 2). Only D265A, S267K, P329G and P329A mutations eliminated binding under the CD3-BSA PC binding condition (Figure 3B and Table 2).

Given the inability to distinguish C1q binding among the variants in the panel by SPR, as with the cell-based FcγR binding assay, a more sensitive FACS-based experiment was developed. In brief, the antibody is bound to the biotinylated CD3 peptide immobilized to SA Dyna Beads, followed by incubation with C1q alone and C1q together with human complement (C3) [59]. C1q was detected with anti-human C1q APC secondary, and C3 was detected with anti-human C3 PE (BD Biosciences). The assay identified D270A, K322A, P329G, P329G, P331S, and S267K as having the largest impact on the reduction in binding to C1q (Figure 3C,D), consistent with published observations [29].

As with the lower hinge-only variants, no impact on binding to CD3δε was observed by Octet BLI (Table S2). In addition, the retention of binding to ProA was preserved for the entire panel (Table S2).

Biophysical properties were profiled by PSR, AC-SINS, HIC, DSF Tm, SEC, and pH 3.5 stress SEC (Table 3). The PSR variation among the panel was 0.33 (WT)–0.43 (LALA-L309A). The HIC retention time was unchanged for the entire panel, implying that hydrophobicity was not impacted. Increases in self-interaction propensity assessed by AC-SINS were evident for LALA combinations with D270A and L309V, and LALAGA-P329G, which yielded maximal wavelength shifts more than double that of WT. Overall there was somewhat greater variation among the combination panel than for lower hinge substitutions alone.

**Figure 4:**
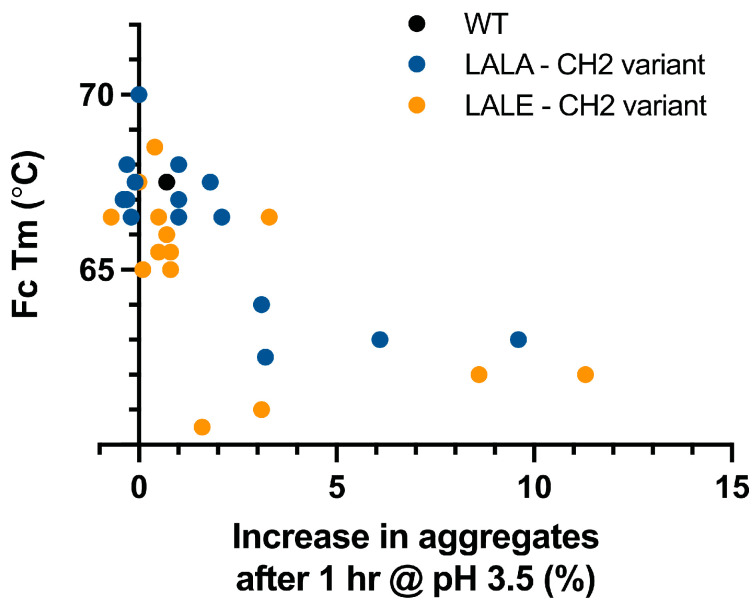
Destabilization of the Fc CH2 domain is associated with increased aggregation propensity at low pH. A CH2 mutant panel was paired with LALA and LALE lower hinge mutations. Fc Tm was determined as the lowest melting event observed by DSF, which for WT human IgG1 CH2 is ~67.5 °C. A relationship between reduced CH2 stability and increased susceptibility for forming aggregates at pH 3.5 was observed.

The most significant deviations from WT-like properties were observed for DSF Tm and pH 3.5 stress SEC (Δ% monomer), reflecting changes in stability. The DSF Tm assay reports the lowest value associated with a thermal transition event, attributed to the unfolding of the CH2 domain. WT IgG1 has a Tm of 67.5 °C under the standard conditions of this assay. CH2 mutations associated with the largest reduction in DSF Tm are D265A (63.0 °C), L328A (63.0 °C), and P331S (62.5 °C) in combination with LALA and D265A (62.0 °C), L328A (62.0 °C), P331S (60.5 °C), and A330S/P331S (61.0 °C) in combination with LALE (Table 2). The pH 3.5 stress SEC assay measures aggregation propensity under acidic conditions, reported as Δ% monomer. D265A shows a 9.6% and 11.3% increase in aggregates in combination with LALA and LALE, respectively, compared with WT (Table 2 and Figure S2). L328A, P331S, and A330S/P331S also show increases in aggregates ranging from 3.1–8.6 when combined with LALA and LALE. Thus, variants that significantly reduced CH2 Tm generally also showed increased propensity to form aggerates under acidic conditions (Figure 4 and Table 3). The single exception is LALE-P331S, which forms fewer aggregates (1.6% increase over WT) than LALA-P331S (3.2% increase over WT).

## 4. Discussion

IgG1 antibodies that display biophysical properties similar to WT while possessing silenced effector function have clear benefits for the manufacturing process. One key aspect of this is resistance to acid-induced aggregation, which can become an issue during the low pH hold routinely employed for viral inactivation [60]. The alternative use of detergent is less straightforward and may affect yield to a greater extent than low pH [61]. While stabilizing mutations for IgG4 are known [62], IgG1 has significantly higher representation in marketed therapeutic antibodies; approximately 59% are IgG1 whereas IgG2 and IgG4 comprise 7% and 21%, respectively [63]. This may reflect a more straightforward manufacturing process and the favorable developability and functional profile of IgG1.

LALA-P329G is among the most prevalent mutation sets in clinical use, represented by alnuctamab, cergutuzumab, cibisatamab, glofitamab (approved), simlukafusp, melredableukin, englumafusp, eciskafusp, tobemstomig, lomvastomig, forimtamig, and simaravibart. In contrast to LALA-P329G, LALA-P329A has not been well studied and the specific combination LALE-P329G in this work has not been previously described. A study reporting the effect of introducing LALA, P329G, and P329A mutations examined the combination of LALA with P329G but not with P329A, based on the rationale that P329G and P329A could be distinguished under certain conditions [35]. Using both SPR and cell-based assays, including highly avid formats, we found that LALA-P329A and LALE-P329G perform very similarly to LALA-P329G in terms of completely abolishing effector function, under the conditions of the assays tested here, with the retention of stability and biophysical developability profile. LALA-S267K and LALE-S267K also appear to work quite well. In contrast, while LALA-D265A, LALE-D265A, LALA-P331S, and LALE-P331S show reduced Fc (CH2) Tm and increased propensity for acid-induced aggregation. This finding is consistent with another study that demonstrated reduced stability and expression for the D265A variant [64]. Moreover, while D265A is effective in abrogating the binding of FcγR, it is not effective for the elimination of the binding of C1q (Figure 3D).

Another promising mutation set, L234G, L235T, and G236R (LGLS-G236R), was recently described and could not be included in our study [65]. Binding to FcRn, FcγR, and C1q was characterized, including both in vitro and in vivo assays; however, biophysical characterization was limited to SEC at neutral pH and thermal stability.

In summary, this work explores the impact on biophysical properties of commonly used antibody constant domain variants that reduce or eliminate ADCC and CDC effector functions while retaining the ability to bind FcRn and ProA. Our findings may be extended by cell-based functional data, as it is possible that our in vitro assays may not be completely predictive of the ability to engage FcγR and C1q under in vivo conditions. However, the majority of mutations have been subjected to such assays, either individually or in some combinations (e.g., LALA-P329G), and it is unlikely that the functional properties of the newly generated mutations sets would fall outside of expectations based on these historical assessments.

## Figures and Tables

**Table 1:** Lower hinge mutation set characterization.

Mutations	Hinge Sequence	Isotype	PSR ^1^ (Score)	AC-SINS (Δmaxλ)	HIC RT (min)	SEC (%)	pH 3.5 Stress SEC (%)	Fc Tm (°C)	THP-1 (MFI)
N/A	PAPELLGG	WT IgG1	0.27	7.7	8.0	97.7	97.6	67.5	7136
L234A/L235A (LALA)	PAPE**AA^2^**GG	IgG1	0.28	8.1	8.0	98.1	97.1	68.5	491
L234F/L235E (LFLE)	PAPE**FE**GG	IgG1	0.28	6.9	8.0	97.5	98.1	66.0	369
L234A/L235E (LALE)	PAPE**AE**GG	IgG1	0.28	8.4	8.0	98.4	97.9	66.0	192
L235A/G237A (LAGA1)	PAPEL**A**G**A**	IgG1	0.27	7.6	8.0	98.4	97.3	67.0	192
L234A/G237A (LAGA2)	PAPE**A**LG**A**	IgG1	0.28	7.4	8.0	98.5	98.5	67.0	184
L234A/L235A/G237A (LALAGA)	PAPE**AA**G**A**	IgG1	0.29	7.6	8.0	98.2	96.7	67.5	150
L234A/L235K (LALK)	PAPE**AK**GG	IgG1	0.28	7.7	8.0	97.5	81.3	70.5	140
L234F/L235K (LFLK)	PAPE**FK**GG	IgG1	0.28	8.0	8.0	98.1	62.5	70.5	386
L234E/L235K (LELK)	PAPE**EK**GG	IgG1	0.28	7.9	8.0	97.5	79.3	68.5	176
N/A	PAP**PVA-**G	WT IgG2	0.38	10.0	8.0	96.1	97.8	68.0	565

^1^ PSR utilized the homogenized solubilized membrane preparation from CHO-S cells, according to Xu et al., 2013 [45]. ^2^ Bold red text represents change with respect to WT sequence.

**Table 2:** Lower hinge combination panel with CH_2_ mutations’ characterization of binding to FcRn, FcγRI (CD64), FcγRII (CD32a), FcγRIII (CD16), and C1q.

	Biacore Kinetics					FACS Cell Binding		
IgG1 Hinge × CH2 Mutations	FcRn (RU)	CD64 (RU)	CD32a (RU)	CD16 (RU)	C1q (RU)	THP-1 (MFI)	THP-1 CD3-BSA PC (MFI)	C1q Dynal FACS (MFI)
N/A	40.0	92.0	4.8	9.8	155.7	11,755.2	23,338.7	3505.0
LALA	44.0	23.5	0.1	0.6	−13.9	269.6	5274.1	1983.0
LALA-D265A	44.6	1.0	0.0	−0.3	−9.1	113.1	119.2	1948.0
LALA-S267K	43.0	1.5	0.0	−0.6	−8.7	119.0	127.5	797.0
LALA-H268Q	44.0	22.9	0.4	0.2	−10.7	202.7	4799.2	2351.0
LALA-D270A	43.6	9.7	0.1	−0.5	−14.4	131.5	2274.4	769.0
LALA-L309A	27.0	21.6	0.2	−0.5	3.0	168.3	4787.3	2519.0
LALA-L309V	43.3	25.2	0.1	0.2	−2.3	177.8	4846.6	2143.0
LALA-K322A	38.1	19.9	0.1	−0.4	−20.9	171.8	4387.4	875.0
LALA-A327G	42.2	14.3	0.2	0.1	0.0	141.6	7006.4	3146.0
LALA-L328A	40.9	13.6	0.3	0.0	−1.7	149.1	6424.2	1492.0
LALA-P329A	45.1	2.2	−0.1	−0.5	−3.3	121.7	168.9	621.0
LALA-P329G	44.5	2.0	−0.1	−0.3	−25.1	122.2	146.2	676.0
LALA-Y296F	41.9	19.1	0.2	−0.1	−3.2	150.9	3808.6	2077.0
LALA-A330S	43.2	19.9	0.3	0.2	−2.9	175.3	4438.2	1703.0
LALA-P331S	40.7	17.7	0.0	0.0	−22.1	167.5	4215.4	724.0
LALA-A330S/P331S	37.0	15.8	0.0	−0.1	−5.3	163.8	3840.6	813.0
LALE	38.9	3.8	0.1	0.4	−27.3	126.6	5298.4	1787.0
LALE-D265A	36.8	0.5	0.0	−0.4	−16.4	125.3	115.4	2279.0
LALE-S267K	40.1	0.7	−0.1	−0.2	−11.2	121.8	130.3	911.0
LALE-H268Q	39.2	4.3	0.1	0.6	5.7	125.7	4000.1	1934.0
LALE-D270A	40.1	1.8	0.0	−0.1	−19.6	119.7	545.8	885.0
LALE-L309A	26.0	4.2	0.1	0.5	−11.7	124.1	2758.1	2312.0
LALE-L309V	40.9	4.9	0.1	0.6	2.3	122.7	3543.8	1840.0
LALE-K322A	32.4	4.6	0.0	0.0	−4.9	120.7	2323.0	586.0
LALE-L328A	38.8	3.5	0.4	0.5	−6.6	130.2	7040.7	1562.0
LALE-P329G	38.1	0.8	−0.1	−0.1	−23.3	120.3	123.7	660.0
LALE-P329A	37.3	0.8	0.0	−0.3	−6.5	117.5	123.6	865.0
LALE-A330S	38.6	3.3	0.1	0.3	−6.2	125.4	2259.5	1620.0
LALE-P331S	38.6	3.3	0.2	0.2	−120.6	128.6	1762.6	725.0
LALE-A330S/P331S	35.5	2.6	−0.1	0.0	−21.0	123.0	1388.0	750.0
LALAGA-P329G	37.1	0.4	−0.1	−0.3	−6.2	115.1	129.0	664.0

**Table 3:** Lower hinge combination panel with CH2 mutations’ biophysical developability characterization.

IgG1 Hinge × CH2 Mutations	PSR (Score)	AC-SINS (Δmaxλ)	HIC RT (min)	SEC (%)	SEC Aggregate after 1 h at pH 3.5 (%)	Fc Tm (°C)
N/A	0.33	8.1	8.5	97.6	0.7	67.5
LALA	0.37	9.1	8.4	93.8	−0.2	66.5
LALA-D265A	0.40	6.9	8.4	96.1	9.6	63.0
LALA-S267K	0.39	14.6	8.4	94.9	−0.4	67.0
LALA-H268Q	0.40	12.6	8.4	96.3	−0.3	68.0
LALA-D270A	0.41	17.3	8.4	96.4	1.0	68.0
LALA-L309A	0.43	15.0	8.3	95.3	0.0	70.0
LALA-L309V	0.42	17.4	8.3	96.5	1.8	67.5
LALA-K322A	0.38	13.9	8.4	96.0	2.1	66.5
LALA-A327G	0.38	9.7	8.4	98.0	−0.3	67.0
LALA-L328A	0.37	10.8	8.4	96.2	6.1	63.0
LALA-P329A	0.38	10.8	8.4	94.9	−0.4	67.0
LALA-P329G	0.38	12.8	8.4	98.0	1.0	66.5
LALA-Y296F	0.40	12.6	8.4	96.1	−0.1	67.5
LALA-A330S	0.37	11.9	8.4	96.3	1.0	67.0
LALA-P331S	0.39	14.7	8.4	96.3	3.2	62.5
LALA-A330S/P331S	0.37	13.7	8.4	92.8	3.1	64.0
LALE	0.37	11.2	8.4	96.6	0.5	66.5
LALE-D265A	0.38	9.3	8.4	96.0	11.3	62.0
LALE-S267K	0.36	8.0	8.4	97.8	3.3	66.5
LALE-H268Q	0.35	9.6	8.4	97.8	−0.7	66.5
LALE-D270A	0.35	12.7	8.4	96.1	0.0	67.5
LALE-L309A	0.36	12.8	8.3	96.3	0.4	68.5
LALE-L309V	0.39	13.4	8.3	95.4	0.5	65.5
LALE-K322A	0.33	13.6	8.4	95.7	0.1	65.0
LALE-L328A	0.36	8.4	8.4	94.1	8.6	62.0
LALE-P329G	0.36	11.3	8.4	97.4	0.7	66.0
LALE-P329A	0.35	10.5	8.4	96.5	0.8	65.0
LALE-A330S	0.35	13.5	8.4	96.9	0.8	65.5
LALE-P331S	0.35	12.8	8.4	93.9	1.6	60.5
LALE-A330S/P331S	0.35	12.9	8.4	94.5	3.1	61.0
LALAGA-P329G	0.38	16.4	8.4	96.8	0.6	65.0

## Data Availability

The data used to support the findings of this study can be made available by the corresponding author upon request.

## Supplementary Materials

The following supporting information can be downloaded at: https://www.mdpi.com/article/10.3390/antib12030054/s1. Figure S1: Profiling the cell surface expression of FcγRI and FcγRII on THP-1 cells; Figure S2: Representative SEC profiles for WT IgG1 and LALE-D265A variants under standard (PBS) and pH 3.5 conditions; Table S1: Octet BLI characterization of the binding of the lower hinge panel to Cy CD3δε; Table S2: Octet BLI characterization of the binding of the lower hinge CH_2_ mutation combination panel to ProA, Hu CD3δε, and Cy CD3δε.
