# Supplementary material for: Profiling the Biophysical Developability Properties of Common IgG1 Fc Effector Silencing Variants
Source: Antibodies (Basel). 2023 Aug 22;12(3):54. doi: 10.3390/antib12030054 (PMC10526015; doi:10.3390/antib12030054)
Supplement: Supplementary file 1 [file antibodies-12-00054-s001.zip › antibodies-2525521-supplementary.pdf]

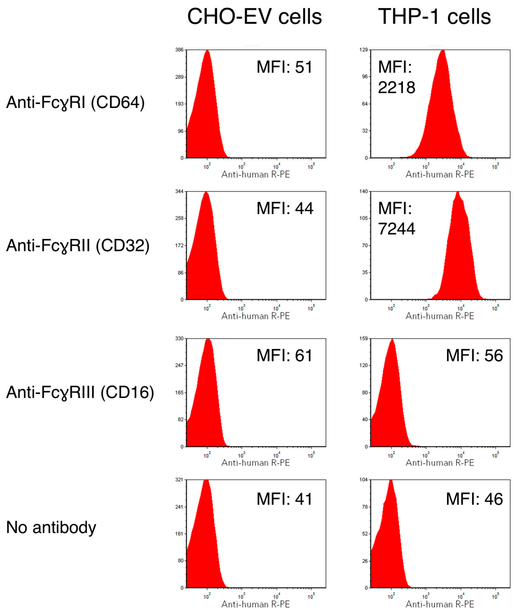

Figure S1. Profiling cell surface expression of FcγRI and FcγRII on THP-1 cells. Anti-FcγRI (CD64) clone 10.1 (BD Biosciences), anti-FcγRII (CD32) clone IV.3, and anti-FcγRIII (CD16) clone 3G8 were used to label the empty vector transfected CHO cells (CHO-EV) and THP-1 cells.

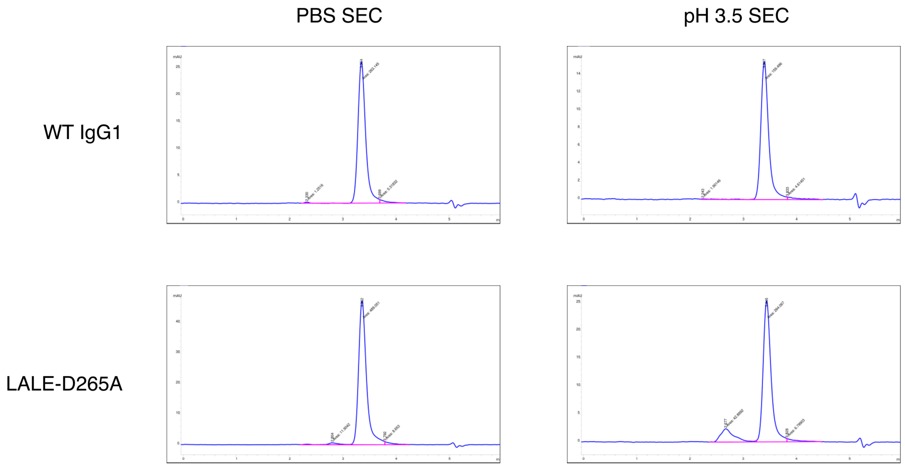

Figure S2. Representative SEC profiles for WT IgG1 and LALE-D265A variants under standard (PBS) and pH 3.5 conditions.

Table S1. Octet BLI characterization of the binding of the lower hinge panel to Cy CD3δε.

IgG1 Hinge Mutations	Cy CD3δε Fc (RU)
WT IgG1	0.47
L234A/L235A (LALA)	0.55
L234F/L235E (LFLE)	0.55
L234A/L235E (LALE)	0.56

L235A/G237A (LAGA1)	0.53
L234A/G237A (LAGA2)	0.52
L234A/L235A/G237A (LALAGA)	0.55
L234A/L235K (LALK)	0.53
L234F/L235K (LFLK)	0.56
L234E/L235K (LELK)	0.59
WT IgG2	0.51

Table S2. Octet BLI characterization of the binding of the lower hinge CH2 mutation combination panel to ProA, Hu CD3δε, and Cy CD3δε.

	Octet Kinetics		
**IgG1 Hinge x CH2 Mutations**	**ProA (RU)**	**Hu CD3δε Fc (RU)**	**Cy CD3δε Fc (RU)**
N/A	4.6	0.52	0.45
LALA	4.9	0.59	0.54
LALA-D265A	4.7	0.51	0.59
LALA-S267K	4.9	0.45	0.57
LALA-H268Q	4.8	0.51	0.59
LALA-D270A	5.0	0.43	0.57
LALA-L309A	4.8	0.45	0.59
LALA-L309V	4.6	0.45	0.60
LALA-K322A	4.8	0.44	0.55
LALA-A327G	4.7	0.54	0.57
LALA-L328A	4.9	0.59	0.59
LALA-P329A	4.9	0.56	0.61
LALA-P329G	4.8	0.53	0.60
LALA-Y296F	4.9	0.47	0.59
LALA-A330S	5.0	0.56	0.59
LALA-P331S	5.0	0.57	0.61
LALA-A330S/P331S	4.7	0.53	0.58
LALE	5.1	0.56	0.50
LALE-D265A	4.8	0.59	0.63
LALE -S267K	4.8	0.55	0.49
LALE-H268Q	5.1	0.52	0.48
LALE-D270A	4.9	0.52	0.48
LALE-L309A	4.7	0.53	0.49
LALE-L309V	0.0	0.56	0.51
LALE-K322A	4.6	0.53	0.51
LALE-L328A	4.7	0.55	0.49
LALE-P329G	5.0	0.55	0.49
LALE-P329A	4.9	0.57	0.52
LALE-A330S	5.1	0.54	0.48
LALE-P331S	5.1	0.55	0.47
LALE-A330S/P331S	5.0	0.58	0.51
LALAGA-P329G	5.0	0.56	0.43
